# Mobile health management among end stage renal disease patients: a scoping review

**DOI:** 10.3389/fmed.2024.1366362

**Published:** 2024-07-11

**Authors:** Yue Wen, Yi Ruan, Yang Yu

**Affiliations:** Department of Nephrology, West China Hospital, Sichuan University, Chengdu, China

**Keywords:** end-stage renal disease, mobile health management, MHealth management, ESRD, MHealth

## Abstract

**Aims:**

The health management of end-stage renal disease patients is a complicated process, and mobile health management technology provides a new choice for the health management of end-stage renal disease patients. The scope of clinical studies on mobile health management for patients with end-stage renal disease was reviewed, and found that about mobile health management problems existing in the literature were identified to provide ideas for subsequent mobile health management research.

**Methods:**

The databases Web of Science, PubMed, The Cochrane Library, Embase, CNKI, Wan Fang Data, BMJ, and VIP were systematically searched for studies on Mobile health management among end-stage renal disease in adult and adolescent patients or children undergoing kidney replacement therapy. The search covered the period from the inception of the databases to June 20, 2023. Two independent reviewers conducted the literature screening process. Following eligibility screening, a total of 38 papers were included for data extraction and descriptive analysis.

**Results:**

A total of 38 studies from 14 countries were finally included. The majority of which were interventional trials. The platforms used in these studies included remote monitoring systems, apps, websites, mobile phones or tablets, and social platforms. These platforms provided patients with a wide range of services, including disease management, behavioral intervention, social support, and follow-up care. Most studies focused on patient clinical indicators, patient experience, quality of life, and healthcare costs.

**Conclusion:**

Our findings that mobile health management has been widely used in disease management of end-stage renal disease patients, with rich management content and many evaluation indicators. Future studies should strengthen the evaluation of patients’ mental health, quality of life, and healthcare costs. Additionally, developing a clinical decision support system would enable mobile health management to play a more effective role in end-stage renal disease patients.

## 1 Introduction

As the global population ages at an unprecedented rate, the incidence of chronic diseases is soaring, and chronic kidney disease (CKD) is no exception (1). The more patients suffer from CKD, the more develop end-stage renal disease (ESRD) (2). ESRD is diagnosed when kidney function is insufficient to sustain life without kidney transplantation or dialysis (3), and its prevalence is also rising (4). The treatment of ESRD is complex and demanding, requiring long-term dialysis therapy (hemodialysis or peritoneal dialysis), kidney transplantation, and drug management (5). Patients with ESRD also face a variety of health management challenges, including cardiovascular disease (6) and skin disorders (7), etc.

In contrast to traditional medical models, which require patients go to a hospital for treatment, peritoneal dialysis can now be performed at home, while hemodialysis is typically conducted at a dialysis center via an arteriovenous fistula (which requires months to mature before use), an arteriovenous graft (which can be used in as little as 24 h, depending on the graft material), or a central venous catheter (which can be used immediately, but poses the highest risk of infection). Vascular access is essential for both hemodialysis and peritoneal dialysis, but kidney transplantation remains the preferred treatment for ESRD patients (5).

Mobile health (MHealth) management technology has emerged as a promising method in modern medicine, offering patients more convenient and effective ways to manage their health (8). MHealth leverages mobile devices and other technologies to improve patient engagement, monitoring, outreach, and healthcare services. It is accelerating the modernization of medicine and has been widely adopted for the management of various chronic diseases (9), including diabetes (10), hypertension (11), cancer (12), and others. In this context, MHealth management technology provides a new and innovative approach to the health management of ESRD patients.

However, the health management of ESRD patients is a complex and challenging endeavor, requiring close collaboration between medical institutions, patients (13) and IT services. It’s not just about data protection and data safety, it’s also about how much project funding or budget there is, and bureaucracy is also an obstacle. MHealth management technology, interventions that provide health-related information through telecommunications or other wireless technologies, such as smartphones, tablets (14, 15) telemedicine, can provide patients with more convenient and personalized healthcare solutions, enabling them to better monitor their health status and improve their overall health and quality of life (16). A scope review is an ideal tool to determine the scope or coverage of the body of literature on a given topic and to specify the amount of literature and research available as well as an overview (17). Previous studies that reviewed the scope of lifestyle interventions provided by eHealth in chronic kidney disease found that there is currently insufficient evidence to recommend the implementation of specific lifestyle e-health interventions in the clinical care of people with chronic kidney disease (18), funding or budget problems which gives researchers the direction and focus of future research in chronic kidney disease. Therefore, a scope review on MHealth management for ESRD patients is warranted to better manage ESRD patients.

This study aims to review the scope of mobile health management for patients with end-stage renal disease and to synthesize and analyze relevant domestic and international literature. The application landscape, advantages and disadvantages, development trends, and future prospects of mobile health management technology in ESRD patient management were comprehensively studied and analyzed to provide a reference for the practice of mobile health management for ESRD patients. We envision that mobile health management technology for ESRD patients will continue to develop and improve in the future, providing patients with more convenient and personalized health management services. Simultaneously, we hope that medical institutions and researchers will pay greater attention to the health management of ESRD patients and provide more comprehensive and in-depth support for research and application in this area.

## 2 Methods

In this study, according to PRISMA Extension for Scoping Reviews (PRISMA-SCR) (19) (see Supplementary file 1) and the Arksey and O’Malley (20) framework of mobile health management in patients with end-stage renal disease in the scope of review. All data can be made available upon reasonable request.

### 2.1 Inclusion and exclusion criteria

Inclusion criteria ① Subjects: laboratory confirmed patients with end-stage renal disease, regardless of sex, age. ② Study types: randomized controlled trial, non-randomized controlled trial, cohort study, case–control study. ③ Literature sources: journal papers published in peer-reviewed journals and dissertations.

Exclusion criteria ① Repeated publication, full text is not available.

### 2.2 Search strategy

A systematic paper search was conducted in PubMed, Cochrane Library, CNKI, VIP, Web of Science, EMBASE, BMJ, and Wan fang digital journal full-text database, we included all papers which were listed in the different searching tools until day June 20, 2023. The search strategy was: (end-stage renal disease OR ESRD) AND (telemedicine OR telehealth OR eHealth OR mobile health OR MHealth).

### 2.3 Literature screening and data extraction

Two researchers (WY and RY) rigorously adhered to the inclusion and exclusion criteria for literature screening. First, they reviewed the titles and abstracts of all identified studies, and then carefully read the full text of any studies that potentially met the inclusion criteria. Any disagreements were resolved through discussion or consultation with a third reviewer. Data were extracted from the included studies, including author, publication year, country, study type, sample size, MHealth management interventions, and evaluation indicators, etc.

## 3 Results

### 3.1 Literature search results

**Figure 1:**
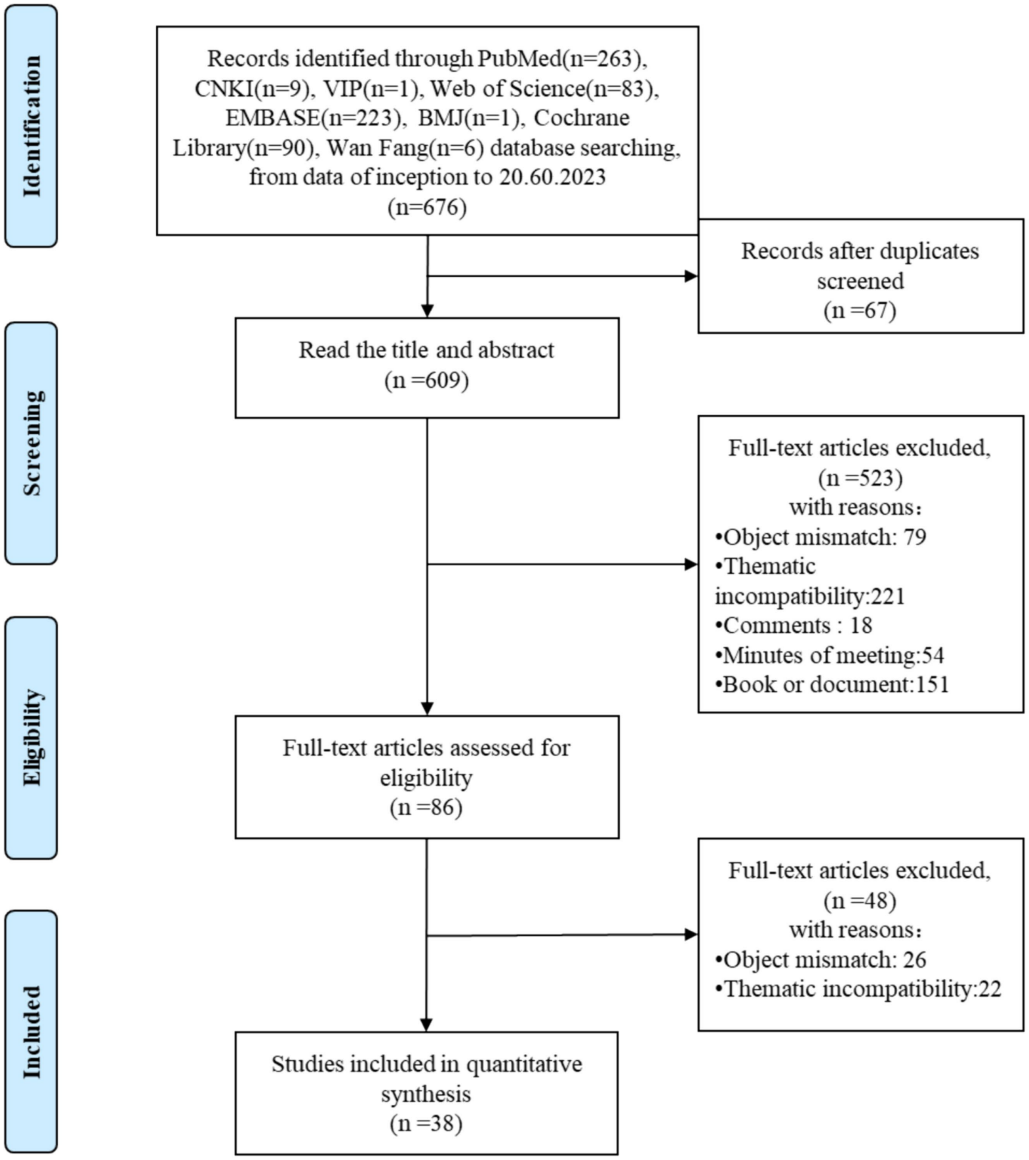
Papers screening process.

A total of 676 papers were retrieved from all databases. After 67 duplicate records were removed, among the remaining 609 relevant studies, 523 were excluded due to being object mismatch, thematic incompatibility, comments, minutes of meeting, book or document. The full text of the remaining 86 studies were read and 48 studies were removed after reading the full text due to object mismatch. The remaining 38 papers were extracted from the corresponding data according to the data extraction requirements. The papers screening process is shown in Figure 1.

### 3.2 Basic information of literature

The 38 included articles were published between 2003 and 2023, originating from 14 countries, with the United States contributing the most (14 articles, 36.84%), followed by China (7 articles, 18.42%) and Denmark (3 articles, 7.89%). In terms of literature types, 16 were randomized controlled trials, 8 were cohort studies, 11 were qualitative studies, and 3 were meta-analyses/systematic reviews, primarily examining ESRD telecare. According to the treatment methods, 11 studies focused on hemodialysis, 9 on peritoneal dialysis, 11 on kidney transplantation, and 7 on unspecified or multiple dialysis methods (Tables 1, 2).

**Table 1:** Basic information of literature.

Type	First author	Study characteristics	Year	Region	Number of analyzed patients	Age (mean/mean ± sd)	Sex (male %)
Hemodialysis	Li Shensen (21)	Randomized Controlled Trial	2012	China	157	61.4 ± 13.3	54.78%
	Zhaohui Ni (22)	Randomized Controlled Trial	2019	China	8,392	60.5 ± 13.7	60.28%
	Raquel Scofano (23)	Qualitative research	2022	Brazil	17	80 ± 20	64%
	Dayna E. Minatodani (24)	Randomized Controlled Trial	2013	USA	99	45–75	55.56%
	Jennifer Gabbard (25)	Randomized Controlled Trial	2021	USA	22	69.4 ± 6.6	36.40%
	Neumann, Claas L (26)	Randomized Controlled Trial	2013	Germany	120	65.7 ± 14.7	/
	Steven J. Berman (27)	Randomized Controlled Trial	2011	USA	44	57–62	54.54%
	Jessica Dawson (28)	Cohort study	2020	Australia	115	/	/
	Eric D. Weinhandl (29)	Cohort study	2017	USA	606	52.4 ± 14.1	35%
	Nicola Elzabeth Anderson (30)	Qualitative research	2021	UK	22	> = 18	/
	Mohsen T orabi Khah (31)	Randomised clinical trial.	2023	Iran	35	45.26 ± 1.42	49%
Kidney transplantation	John W. McGillicuddy (32)	Randomized Controlled Trial	2020	USA	71	52	69%
	Nielsen, Charlotte (33)	Qualitative research	2020	Denmark	/	/	/
	Rachel E. Patzer (34)	Qualitative research	2016	USA	721,339	/	/
	Rachel E. Patzer (35)	Randomized Controlled Trial	2019	USA	470	50.6 ± 10.1	62.50%
	Elisa J. Gordon (36)	Cohort study	2016	USA	63	18–75	/
	A. Schmid (37)	Randomized Controlled Trial	2016	Germany	46	18–66	54.34%
	Edward W. Aberger (38)	Randomized Controlled Trial	2014	USA	66	54	48%
	Lieke Wirken (39)	Qualitative research	2017	Holland	13	58.8 ± 11.5	69%
	Rachel E. Patzer (40)	Randomized Controlled Trial	2016	USA	450	18–75	
	Charlotte Nielsen (41)	Qualitative research	2020	Denmark	4	/	/
	Alfonso M Cueto-Manzano (42)	Qualitative research	2015	México	23	33	57%
Peritoneal dialysis	Zheng Pian (43)	Randomized Controlled Trial	2018	China	107	45.40 ± 8.90	54.21%
	Fu Qiao Hui (44)	Randomized Controlled Trial	2022	China	100	51.96 ± 16.40	53.59%
	Manya Magnus (45)	Randomized Controlled Trial	2017	USA	200	45–64	51%
	Daphne M. Harrington (46)	Cohort study	2014	India	6	24–61	50%
	Tiantian Ma (47)	Cohort study	2022	China	7,000	51.2 ± 14.5	53.70%
	Brett Tarca (48)	Cohort study	2021	Australia	/	/	/
	Vishal Dey (49)	Cohort study	2016	UK	22	61.6	45%
	Giusto Viglino (50)	Qualitative research	2020	Italy	107	72.2 ± 13.1	58.88%
	Xiao Xu (51)	Cohort study	2022	China	7,539	/	/
Not specify the treatment methods	Chi-Sheng Hung (52)	Randomized Controlled Trial	2018	Taiwan, China	715	69.7 ± 11.8	68.00%
	Ji-Eun Kim (53)	Qualitative research	2020	USA	16	58.18 ± 12.67	62.5
	Emily SETO (54)	Qualitative research	2007	Canada	149	52 ± 17	62%
	Abu Bakkar Siddique (55)	Systematic evaluation	2019	USA	/	/	/
	Manuel Prado (56)	Qualitative research	2003	Spain	/	/	/
	Meaghan Lunney (57)	Mate analysis	2018	/	/	/	/
	Priya Ramar (58)	Mate analysis	2017	USA	/	/	/

“/”means not mentioned.

**Table 2:** Characteristics of study interventions and evaluation index.

Type	First author	Intervention duration	Mobile health management measures	Manage the main content or direction	Management mode	Evaluation index
Hemodialysis	Li Shensen (21)	6 months	Monitoring system	Effectiveness of health management	Active management	1. Incidence of dialysis complications; 2. Blood pressure, hemoglobin, blood calcium and phosphorus, blood albumin, standard protein breakdown rate, subjective nutrition score (SGA); 3.Urea clearance index 4. Dialysis adequacy.
	Zhaohui Ni (22)	28 months	Dialysis registration system based on wechat mobile platform	Anemia monitoring	Automatically push	Hemoglobin and hematocrit levels
	Raquel Scofano (23)	6 months	Assisted home hemodialysis	The role of remote monitoring in improving the relationship between doctors, nurses and patients	/	Remote monitoring experience
	Dayna E. Minatodani (24)	42 months	Remote care nurse–patient contact	Health management	Active management	Number of hospital and emergency department visits, length of stay, and total cost of hospital and emergency room services for all patients
	Jennifer Gabbard (25)	6 months	An iPad-based symptom assessment tool	Evaluation feasibility analysis	/	Ease of use of the system
	Neumann, Claas L (26)	3 months	Body weight telemetry	Weight management	Automatic monitoring followed by active management	Interdialytic weight gain
	Steven J. Berman, (27)	12 months	VitelCare Turtle 500	Health management outcomes, quality of life, cost–benefit analysis	Automatic monitoring followed by active management	1. Health outcome measures included hospitalization, emergency room visits, and length of stay. 2. The economic analysis includes total hospital and emergency room costs. 3. Quality of life was measured using the Medical Outcomes Survey tool 36-item Short Form Health Survey (SF-36)
	Jessica Dawson (28)	6 months	Mobile phone short message	Dietary behavior intervention	Automatic SMS push	1. They were measured using recruitment and retention rates, acceptability of the intervention, and adherence to dietary recommendations. 2. Secondary findings included information on certain clinical parameters associated with dietary management in patients receiving maintenance hemodialysis
	Eric D. Weinhandl (29)	1.18 years	Nx2me Interconnect health Platform	To evaluate the mechanisms by which telemedicine platforms improve patient clinical outcomes and patient burden	/	Risk of all-cause attrition, dialysis cessation (i.e., death or transplant) and technical failure
	Nicola Elzabeth Anderson (30)	/	Monitoring system	Evaluate the usefulness of patient-reported outcomes collected by the system	/	/
	Mohsen T orabi Khah (31)	1 month	App	Treatment adherence and perception	Automatically push AND Active management	“Treatment adherence and perception
Kidney transplantation	John W. McGillicuddy (32)	6 months	An electronic medication tray and an mHealth app	Medication adherence intervention	Intelligent reminder	The proportion of patients obtaining normal tacrolimus trough variability
	Nielsen, Charlotte (33)	/	App	Improve follow-up after renal transplantation	/	/
	Rachel E. Patzer (34)	/	A mobile clinical decision aid (iChoose Kidney)	Estimates of risks of death and survival on dialysis compared to kidney transplantation	/	The discriminatory ability of the model for 3-year mortality
	Rachel E. Patzer (35)	12 months	A mobile clinical decision aid (iChoose Kidney)	Improving knowledge about treatment options among transplant candidates	Intelligent reminder	Change in transplant knowledge
	Elisa J. Gordon (36)	3 weeks	A Website	Increase Knowledge About Living Kidney Donation and Transplantation Among Hispanic/Latino Dialysis Patients	/	Participants’ knowledge scores
	A. Schmid (37)	12 months	Telemedicine support	Optimize Routine Evidence-Based Aftercare	Custom management	Medical outcomes, adherence, quality of life and costs
	Edward W. Aberger (38)	6 months	Telemedicine systems and electronic blood pressure monitoring systems	Enhancing Patient Engagement and Blood Pressure Management	Automatically push AND Active management	Systolic, diastolic, and pulse rate
	Lieke Wirken (39)	/	internet	A guided and tailored internet-based cognitive behavioral therapy (ICBT) intervention for donors and donor candidates was developed and the feasibility and perceived effectiveness were evaluated.	Custom management	Health related quality of life, anxiety and depression
	Rachel E. Patzer (40)	8 months	iChoose Kidney	Improve access to individualized prognosis information comparing dialysis and transplantation outcomes	Auxiliary management	1. Change in knowledge; 2. Change in treatment preferences,; 3. Improved decisional conflict, and increased access to kidney transplantation
	Charlotte Nielsen (41)	/	APP	Development of a telehealth solution to improve the kidney transplantation process	Active management	/
	Alfonso M Cueto-Manzano (42)	4 months	Mobile phone short message	Improve lifestyle and adherence of patients	Automatically push AND Active management	The usefulness of the text messages, the medication reminders, the appointment reminders
Peritoneal dialysis	Zheng Pian (43)	6 months	APP	Explore the application effect of mobile medical app in the follow-up management of peritoneal dialysis patients	Automatically push AND Active management	1. Incidence of complications: peritonitis incidence, catheter outlet infection rate, tunnel infection rate, hospitalization rate 2. Daily record of indicator changes: weight. Bmi. Blood pressure, Ultrafiltration, Urine Volume 3. Test indicators: Hemoglobin (Hb), Albumin (Alb), hemoglobin (HB), albumin (ALB),Serum creatinine (Scr), Calcium (Ca), Phosphorus (P), serum Creatinine (SCR),Blood urea nitrogen (BUN), Intact parathyroid hormone (iPTH) and urea clearance (Kt/V) indicators
	Fu Qiao Hui (44)	12 months	Internet Plus cloud platform	Evaluate the management effect of various management modes	Automatically push AND Active management	Clinical data, laboratory test indicators, peritonitis incidence, tube drift incidence, tube blockage incidence, dropout rate, Duration of peritoneal dialysis treatment, average length of stay in patients exiting peritoneal dialysis”
	Manya Magnus (45)	2 times	Specific educational online videos	Understand patient satisfaction with telemedicine	/	Blood pressure, weight, glucose and peritoneal dialysis (PD)-specific educational online videos for ESRD patients using PD
	Daphne M. Harrington (46)	251 days	A Tablet Computer Platform	The Use of a Tablet Computer Platform to optimize the Care of Patients Receiving to assess their usage in a pilot trial	Active management	Compliance with the applications ranged from 51–92%. No major adverse events were recorded. The overall impression of the interface was 5.2 out of 10
	Tiantian Ma (47)	/	The PD telemedicine App called Manburs	To explore potential predictors and their effects on patient survival, technique survival, and the occurrence of infectious and noninfectious complications.	Automatically push AND Active management	Patient survival, technique survival, hospitalization, and the occurrence of infectious and noninfectious complications.
	Brett Tarca (48)	7 days	Ecological momentary assessment mobile application	Explore the real-time relationships between fatigue, mood and physical activity in people with ESKD receiving peritoneal dialysis.	/	Fatigue and mood
	Vishal Dey (49)	15 months	Computer tablets	To explore patient acceptability of technology and evaluate its effect on clinical interventions and quality of life in patients undergoing peritoneal dialysis	Automatically push AND Active management	QUEST and QOL outcomes: Satisfaction scores retention rates Clinical interventions: admissions and supporting patients to self-manage from the comfort of their home.
	Giusto Viglino (50)	19 ± 12.9 months	Video dialysis	To overcome physical, cognitive and psychological barriers to PD.	Active management	Peritonitis incidence Assisted PD patients, with a family member/live-in carer patients selfcare patients
	Xiao Xu (51)	at least 20 months	The Peritoneal Dialysis Telemedicine-assisted Platform and TM app (Manburs)	Aimed to explore the long-term effects of TM on the mortality and technique failure	Automatically push AND Active management	All-cause mortality
Not specify the treatment methods	Chi-Sheng Hung (52)	24 weeks	Internet-based platform	Aimed to evaluate the effect of renal function status on hospitalization among patients receiving this program and to evaluate the relationship between contract compliance rate to the program and risk of hospitalization in patients with CKD	Automatically push AND Active management	1. Contract Compliance Rate to the Telehealth Program 2. Renal Function and Hospitalization 3. Interaction Between Renal Status and Contract Compliance Rate With Telehealth
	Ji-Eun Kim (53)	/	A personalized mobile dialysis device	To examine patients’ and caregivers’ design preferences and feature considerations for an Ambulatory Kidney to Improve Vitality	/	/
	Emily Seto (54)	/	Internet use	To ascertain the prevalence and predictors of Internet use by ESRD patients among different dialysis modalities.	/	The prevalence and predictors of Internet use
	Abu Bakkar Siddique (55)	/	Mobile Apps	To comprehensively evaluate mobile apps used for medication compliance and nutrition tracking for possible use by CKD and ESRD patients	/	Mobile App Rating Scale
	Manuel Prado (56)	/	A novel telehealth care system for ESRD patients called VCRS	/	/	/
	Meaghan Lunney (57)	/	Telephone, telemetry or video conferencing	Systematically reviewed studies that examined the effectiveness of telehealth versus or in addition to usual care for ESRD management	/	/
	Priya Ramar (58)	/	Remote monitoring	Effects of Different Models of Dialysis Care on Patient-Important Outcomes	/	The effect of interventions on mortality and hospitalizations

“/“means not mentioned.

### 3.3 Types of MHealth management

#### 3.3.1 Remote monitoring system

Of the 38 studies included in the review, 13 (34.21%) mentioned remote monitoring systems. The following is a description of these systems by treatment method:

- Hemodialysis: there were a total of 7 literature, four of which assessed the effectiveness of remote monitoring for health management, i.e., the use of a remote monitoring system to track patients’ vital signs, such as blood pressure and weight. Secondly, the monitoring content includes quality of life detection, mainly focusing on the quality of life and mental health of patients; and monitoring the patient’s medical burden, such as medical costs. And two of the studies examined the potential of remote monitoring to improve doctor-patient relationships and the patient-reported outcomes collected through this modality. One of the study just focused on remote weight monitoring.
- Peritoneal dialysis: one study evaluated the management effectiveness of various peritoneal dialysis modalities.
- Kidney transplantation: two studies focused on kidney transplantation, with one focusing on optimizing routine evidence-based aftercare and the other on enhancing patient engagement and blood pressure management.
- Unspecified treatment methods: three studies did not specify the treatment methods used. One study evaluated the impact of hospitalization on patients receiving telemedicine and the relationship between compliance and hospitalization risk. The second study explored the impact of different dialysis nursing modes on patient outcomes.

#### 3.3.2 App

Of the 38 studies included in the review, 9 (23.68%) mentioned mobile app. These studies were categorized by treatment method as follows:

- Hemodialysis: the study focused on changes in patients’ treatment adherence and perception though app and face-to-face training, and the result showed that such improvements were detected much more in the patients trained with APP based on the micro-learning method than face-to- face training.
- Kidney transplantation: three studies focused on kidney transplantation, with one assessing medication adherence and two examining improved follow-up after kidney transplantation.
- Peritoneal dialysis: four studies focused on peritoneal dialysis. Two of these studies, named “Manburs,” explored potential predictors of peritoneal dialysis patients and their effects on patient survival, technical survival, infectious disease occurrence, non-infectious complications, and long-term mortality. The other two studies examined the effectiveness of follow-up management in peritoneal dialysis patients and the real-time relationship between fatigue, mood, and physical activity in ESRD patients undergoing peritoneal dialysis.
- Unspecified treatment methods: one review systematically evaluated the impact of mobile apps on medication adherence and nutrition tracking.

#### 3.3.3 Phone or tablet

Among the 38 literatures, a total of 6 (15.78%) mentioned the use of mobile phones or tablet for health management, as follows:

- Hemodialysis: there were two studies, and one of which, adopted mobile phone short message to intervene eating behavior regularly. Another one analyzed the feasibility of data collected by iPad.
- Kidney transplantation: there was one used mobile phone text messages, regular text messages to improve the patient’s lifestyle and persistence
- Peritoneal dialysis: there were 2 papers used a tablet computer, which mainly focused on the satisfaction and retention rate.
- Unspecified treatment methods: there was one study to adopted mobile phone short message to intervene eating behavior regularly.

#### 3.3.4 Website

- Peritoneal dialysis: one web-based study assessed patient satisfaction with telemedicine.
- Kidney transplantation: five studies used this approach, including one web-based study that disseminated kidney transplantation knowledge and one qualitative study that explored the evaluation of interventions for kidney donors and donor candidates. Three studies used a mobile clinical decision aid called “iChoose Kidney,” which helped patients discuss treatment plans at the onset of ESRD and improved their knowledge of kidney transplantation, which could influence their decision-making.
- Unspecified treatment methods: one study analyzed patients’ internet use.

#### 3.3.5 Social media

For hemodialysis, there has a collection system based on WeChat to monitor anemia.

#### 3.3.6 Other types

One article described the use of video dialysis to train peritoneal dialysis patients, providing them with essential information about PD and improving the quality of their training. Another article reported on the design of a personalized mobile dialysis device to enhance the vitality of dialysis devices.

### 3.4 Content of mobile health management

The overwhelming majority of studies (28 articles, 73.68%) used electronic archives to ascertain baseline information, including age, sex, and geographic region. Further data monitoring and management were conducted based on electronic records.

#### 3.4.1 Disease management

Disease management is primarily reflected in the monitoring of objective indicators, including clinical and physical parameters. Physical parameters include blood pressure, weight, and so on; clinical indicators include laboratory and clinical findings. Specific examples are as follows:

- Hemodialysis: three studies reported on real-time guidance and personalized intervention through disease monitoring. For example, in studies Li Shensen (21) and Minatodani (24), Berman (27), data uploaded to the network in real time through the remote monitoring system were monitored, analyzed, and evaluated by medical staff, who then provided personalized feedback and guidance to patients.
- Peritoneal dialysis: five studies described timely intervention and treatment by medical staff after automatic monitoring.
- Kidney transplantation: seven studies reported on personalized guidance and management by doctors. For example, Nielsen’s (33), Schmid’s (37) and Aberger’s (38) studies proposed allowing consultations via telephone, video, or online, or introducing training courses for patients.
- Unspecified treatment methods: Hung’s (52) study exemplified the automatic monitoring push and personalized guidance of disease management.

#### 3.4.2 Behavioral intervention

Behavioral intervention is primarily reflected in health behaviors, such as medication adherence and dietary compliance. Nine studies reported on behavioral intervention.

- Hemodialysis: two studies focused on healthy behaviors, including weight management monitoring to guide patients in weight control and dietary advice.
- Peritoneal dialysis: three studies mentioned behavioral intervention, such as diet advice and weight management (43, 44, 49).
- Kidney transplantation: three studies evaluated interventions to improve compliance. For example, two studies (32, 42) reported improved medication adherence through remote intervention management, and one study (37) showed a reduction in non-compliance through remote monitoring.
- Unspecified treatment methods: Huang’s study (52) exemplified behavioral intervention, with nurse case managers communicating with patients daily over the phone as needed to ensure medication and medical instruction adherence.

#### 3.4.3 Social support

Social support encompasses patient-clinician communication and question-and-answer sessions, as well as the support provided by clinicians to patients through remote monitoring and behavioral interventions. Notably, Viglino’s study (50) found that video dialysis enhanced patients’ confidence in peritoneal dialysis (PD).

#### 3.4.4 Self-management and reminders

Here are 10 articles on follow-up management, and they are distributed across different treatment modalities. For hemodialysis, there are three studies mentioned follow-up management (21, 24, 27), for peritoneal dialysis, there are four studies mentioned follow-up management (43, 44, 47, 51), for kidney transplantation, follow-up management was mentioned in two studies (38, 40), for unspecified treatment methods, One study mentioned follow-up management (52). Follow-up management and reminders primarily involve the regular monitoring and communication with patients to assess their overall self-management behavior, provide medication reminders, dietary guidance, and exercise guidance, and schedule follow-up appointments. Ten studies reported using the internet, mini-programs, phone calls, or text messages for follow-up management and reminders.

### 3.5 Evaluation index of health management

#### 3.5.1 Clinical indicators

A diverse range of studies have investigated the clinical indicators associated with m-health management. The main indicators include:

- Dialysis indicators: dialysis adequacy
- Complication indicators: complication rate
- Daily recording indicators: body weight, BMI, blood pressure, ultrafiltration, urine volume
- Assay parameters: hemoglobin, albumin, calcium, phosphorus, serum creatinine, blood urea nitrogen, intact parathyroid hormone (iPTH), urea clearance
- Medical indicators: length of hospital stay, average length of hospital stay, number of emergency department visits, treatment duration
- Survival situation indexes: survival rate, survival time, life expectancy.

#### 3.5.2 Quality of life indicators

Two studies assessed the impact of MHealth management on quality of life.

In hemodialysis, Berman (27) used the 36-item Short Form Health Survey (SF-36) to measure quality of life. In peritoneal dialysis, Dey (49) assessed quality of life before and after a MHealth management intervention.

#### 3.5.3 Cost index

For hemodialysis, Berman (27) Economic analysis of total hospital and emergency room costs; for kidney transplantation, Schmid (37) involved the reduction of nursing cost.

#### 3.5.4 Patient experience

Among the included studies, 7 (18.42%) assessed patient experience and satisfaction, including the availability of remote monitoring systems, apps, or professional websites, and the feasibility of MHealth management measures.

- Hemodialysis: one study (55) evaluated the patient experience of remote monitoring, one study (25) assessed the system’s usability, and one study (30) evaluated the system’s role in collecting patient reports.
- Peritoneal dialysis: one study (45) analyzed the satisfaction of nurses with remote monitoring, one study (46) assessed the satisfaction with the interface, and one study (49) evaluated the satisfaction with remote assistive technology.
- Unspecified treatment methods: one study (53) found that research is conducive to improving the efficiency, effectiveness, and user satisfaction of AKTIV prototypes and products.

### 3.6 Other

For example, four studies (35, 36, 39, 42) assessed the acquisition of transplanted knowledge, and one study (48) investigated the real-time relationships between fatigue, mood, and physical activity in people with ESRD receiving peritoneal dialysis.

## 4 Discussion

The scope review commences with an examination of MHealth management types, MHealth management content, MHealth management evaluation indices, and other relevant aspects. The studies included in this review utilized various platforms such as remote monitoring systems, apps, websites, mobile phones or tablets, and social platforms to offer patients a wide array of services encompassing disease management, behavioral intervention, social support, and follow-up care. These studies primarily focused on patient clinical indicators, patient experience, quality of life improvements, and healthcare cost. It is discussed from the following aspects.

### 4.1 MHealth has been widely used in ESRD patients

This study found that the volume of literature on MHealth management for ESRD has steadily increased since 2003, reflecting the growing convergence of mobile internet technology and medicine. In the early stages, patient management was primarily conducted via phone, text messages, and other simple modalities. However, in recent years, research has focused on developing app-and mini-program-based interventions that leverage the internet and monitoring systems to facilitate personalized interventions based on automatically uploaded health data and automated push or early warning notifications. This study demonstrates that MHealth management has been widely adopted in kidney disease management, with a diverse range of applications. Similarly to the review (18), both demonstrate the breadth of e-health interventions used to provide lifestyle interventions in the CKD population.

### 4.2 MHealth has obvious advantages in ESRD patients

Overall, MHealth offers several advantages for ESRD patients, enabling comprehensive multi-platform management from the dissemination of relevant knowledge to the monitoring of physiological parameters and disease intervention. MHealth also facilitates effective doctor-patient communication. We analyzed different treatment methods separately. From the perspective of mobile management carrier types, hemodialysis research is relatively comprehensive. In terms of research content, the peritoneal dialysis system in China, a telemedicine-assisted platform and telemedicine app (47, 51), has demonstrated promising results in a large-sample cohort study, which was real-world associations between telemedicine use and reduced survival and technology survival in peritoneal dialysis patients. Among kidney transplantation methods, iChoose Kidney (34, 35, 40) from the United States is a prominent mobile health management platform that not only provides transplant-related knowledge, but also predicts the mortality risk of dialysis and transplantation, and aids decision-making for kidney transplantation. Notably, iChoose Kidney offers outstanding functional features, but lacks follow-up management after kidney transplantation, while other MHealth management functions are relatively basic, focusing on the dissemination of transplant knowledge. From the perspective of evaluation indicators, most studies focus on clinical and patient experience indicators. We observed that most studies paid more attention to the physical health status of patients, with vital signs and kidney function being the primary monitoring indicators and evaluation outcomes. Additionally, we found that most studies monitored the health data of ESRD patients through mobile health management, and background medical staff analyzed and evaluated the data, providing further interventions for patients with abnormal conditions, such as adjusting medication or recommending outpatient clinic visits.

### 4.3 Several existing problems of MHealth in ESRD patients

#### 4.3.1 Lack of research on social media platforms

The application of peritoneal dialysis and kidney transplantation lacks research on social media platforms, which are commonly used and familiar to us. Strengthening the interaction with social media platforms could enhance effective communication between medical staff and patients, improve patient engagement, and boost management efficiency.

#### 4.3.2 Single-sample management

Single-sample management studies are still present in hemodialysis, such as those that monitor and manage only weight (26) or anemia (22).

#### 4.3.3 Lack of evaluation of quality-of-life and cost indicators

Further research is needed to determine whether mobile health management can improve patients’ quality of life and reduce costs.

ESRD can seriously affect patients’ quality of life (59). It involves a variety of physical and emotional challenges, including frequent medical interventions, dialysis treatments, dietary restrictions, and limitations in daily activities. In this context, MHealth applications can play a key role in providing personalized care, symptom management and support. By integrating these technologies into the healthcare ecosystem, patients can better self-manage, reduce hospital admissions, and improve overall well-being. ESRD and its associated treatments, such as dialysis and transplantation, can be a financial burden on individuals and healthcare systems (60). Given the long-term nature of ESRD management, cost-effectiveness is an important issue. MHealth applications have the potential to optimize the allocation of medical resources, streamline care processes, and reduce unnecessary expenses. By allowing patients to actively participate in the medical process, these technologies enable more efficient use of resources, resulting in cost savings for patients and providers.

#### 4.3.4 Mental health is rarely considered in management

Depression is the most common psychiatric disorder in patients with ESRD, with a prevalence of 22.8 to 39.3% in the dialysis population (61). However, in this study, few people paid attention to mental health, and we found that the psychological management of mobile health management is becoming more and more abundant. For example, Chou’s research (62) found that chatbots can promote the mental health of the elderly and reduce depressive symptoms. Therefore, integrating it into mobile health management and offering enhanced psychological support represents a key future direction for mobile health management in ESRD patients.

#### 4.3.5 Lack of a big data-driven clinical decision intervention system

These interventions are rarely based on big data decision support systems, lack accurate evidence-based feedback, and lack clinical decision support. However, sometimes data security and privacy concerns affect the development of decision support systems (63). Clinical decision support refers to the integration of electronic medical records and other clinical information through computer technology, automatic processing of patient data, and intelligent medical and nursing recommendations to provide the best plan to help patients make the best clinical decision (64). Clinical decision support is used in the management of many chronic diseases, such as hypertension (65) and advanced heart failure (66). In this study, only the “iChoose Kidney” tool in the kidney transplantation domain exhibited decision-making capabilities. There are studies of biomedical based remote diagnosis of kidney disease, for example, electrochemical creatinine (Bio) sensors for point-of-care diagnosis of renal malfunction and CKD (67). Studies have also been conducted through the development and validation of mixed Brillouin-Raman spectroscopy for non-contact assessment of the mechanochemical properties of urinary proteins as biomarkers for kidney disease (68). Therefore, developing a mobile management decision support system with diversified functions to provide optimal clinical decision support to patients is a key area for future development, and based on biomedical remote instant of ESRD disease diagnosis is also worth exploring, In the context of ESRD, clinical decision support systems can help healthcare providers make timely and informed decisions about treatment choices, medication management, and care planning. By integrating patient-specific data from mobile health apps, clinical decision support systems can enhance clinical decision making, improve treatment outcomes, and potentially reduce the occurrence of medical errors. And how to develop and adopt a standardized set of evaluation metrics and evaluation methods to compare different MHealth applications and platforms. This may include validation based on assessments of enablement and functionality, user satisfaction surveys, and clinical trials.

#### 4.3.6 Lack of other patients MHealth management support, such as patients with disabilities or the elderly (digital newbies) or poor people

At the same time, there is a lack of relevant research on MHealth management for special ESRD population, especially applicability and accessibility of MHealth applications. This may include specific features and interface designs for these patients to ensure they can easily use these apps. Also can consider exploring how assistive technologies and technical support can be used to help these patients overcome barriers to use. And, how the elderly (digital newbies) or individuals from low-income backgrounds can access and benefit from MHealth treatments is also a question. This entails providing technical training and support, enhancing digital engagement capabilities, and improving accessibility to devices and networks. Simultaneously, there is a need to increase public policies and initiatives to ensure that individuals from low-income backgrounds have equitable access to necessary medical treatment and support.

## 5 Conclusion

Following the scoping review reporting framework of Arksey and O’Malley (20), this study reviewed relevant studies on MHealth management for ESRD patients to synthesize the types, contents, and evaluation indicators of MHealth management. The findings revealed that MHealth management has been widely adopted in the disease management of ESRD patients, encompassing a diverse range of management content and numerous evaluation indicators. Future research should focus on enhancing the evaluation of patients’ mental health, quality of life, and costs, as well as developing a clinical decision support system to better realize the potential of MHealth management in ESRD patients.

## 6 Limitation

There was a suggestion at the June 2019 Consensus meeting on Improving Global Results in Kidney Disease (KDIGO) to use “kidney failure” and appropriately describe whether symptoms, signs and treatments are present, rather than “end-stage kidney disease, “but since it is limited to English (nuances can be difficult to translate) (69), Therefore, “end-stage renal disease” was still used for the search in this review, resulted in 38 number of papers in this scope review. Additionally, this scope review starts from types of MHealth management, content of mobile health management, evaluation index of health management, and Others four aspects have been reported, lack of the responsibility of the government to establish modern medicine, including MHealth products and other aspects of sorting. Lastly, quality assessment of included studies is not a primary component of a scoping review (18), therefore critical appraisal is not provided.

## Author contributions

YW: Writing – original draft, Writing – review & editing. YR: Writing – original draft, Writing – review & editing. YY: Writing – original draft, Writing – review & editing.
